# Effects of Curcumin on the Proliferation and Mineralization of Human Osteoblast-Like Cells: Implications of Nitric Oxide

**DOI:** 10.3390/ijms131216104

**Published:** 2012-11-29

**Authors:** Jose M. Moran, Raul Roncero-Martin, Francisco J. Rodriguez-Velasco, Julian F. Calderon-Garcia, Purificacion Rey-Sanchez, Vicente Vera, Maria L. Canal-Macias, Juan D. Pedrera-Zamorano

**Affiliations:** Metabolic Bone Diseases Research Group, School of Nursing and Occupational Therapy, University of Extremadura, Caceres 10003, Spain; E-Mails: jmmorang@unex.es (J.M.M.); rronmar@unex.es (R.R.-M.); fcorodriguezv@unex.es (F.J.R.-V.); jfcalgar@unex.es (J.F.C.-G.); prey@unex.es (P.R.-S.); viventevera@odon.ucm.es (V.V.); luzcanal@unex.es (M.L.C.-M.)

**Keywords:** osteoblast, nitric oxide synthase, nitric oxide

## Abstract

Curcumin (diferuloylmethane) is found in the rhizomes of the turmeric plant (*Curcuma longa* L.) and has been used for centuries as a dietary spice and as a traditional Indian medicine used to treat different conditions. At the cellular level, curcumin modulates important molecular targets: transcription factors, enzymes, cell cycle proteins, cytokines, receptors and cell surface adhesion molecules. Because many of the curcumin targets mentioned above participate in the regulation of bone remodeling, curcumin may affect the skeletal system. Nitric oxide (NO) is a gaseous molecule generated from l-arginine during the catalization of nitric oxide synthase (NOS), and it plays crucial roles in catalization and in the nervous, cardiovascular and immune systems. Human osteoblasts have been shown to express NOS isoforms, and the exact mechanism(s) by which NO regulates bone formation remain unclear. Curcumin has been widely described to inhibit inducible nitric oxide synthase expression and nitric oxide production, at least in part via direct interference in NF-κB activation. In the present study, after exposure of human osteoblast-like cells (MG-63), we have observed that curcumin abrogated inducible NOS expression and decreased NO levels, inhibiting also cell prolifieration. This effect was prevented by the NO donor sodium nitroprusside. Under osteogenic conditions, curcumin also decreased the level of mineralization. Our results indicate that NO plays a role in the osteoblastic profile of MG-63 cells.

## 1. Introduction

Curcumin ((1E,6E)-1,7-bis(4-hydroxy-3-methoxyphenyl)-1,6-heptadiene-3,5-dione or diferuloylmethane) is found in the rhizomes of the turmeric plant (*Curcuma longa* L.), a perennial herb belonging to the ginger family that is cultivated extensively in south and southeast tropical Asia [1]. Turmeric has been used for centuries as a dietary spice and as a traditional Indian medicine used to treat anorexia, rheumatism, sinusitis, hepatic disorders and inflammation [2]. It has also been recognized for its antiproliferative properties in cancer treatment [1]. In particular, curcumin has been shown to regulate the expression of genes implicated in cell proliferation, metastasis, chemotherapy resistance and angiogenesis [1,3]. The anti-neoplastic properties of curcumin have been exhibited in many types of malignancies, including breast cancer, colon cancer, kidney cancer, leukemia, prostate cancer, melanoma and osteosarcoma [1,4]. However, the potential of curcumin has not been systematically examined through multi-center, randomized, double-blind, placebo-controlled clinical trials [1].

At the cellular level, curcumin modulates important molecular targets: transcription factors (such as nuclear factor-κB, activating protein-1, β-catenin and peroxisome proliferator-activated receptor-γ), enzymes (cyclooxygenase-2,5-lipoxygenase and inducible nitric oxide synthase), cell cycle proteins (cyclin D1 and p21), cytokines (tumor necrosis factor-α, interleukin-1, interleukin-6 and chemokines), receptors (epidermal growth factor receptor, low-density lipoprotein receptor, estrogen receptor-α) and cell surface adhesion molecules [1,5–7]. Because many of the curcumin targets mentioned above participate in the regulation of bone remodeling, curcumin may affect the skeletal system. In fact, the effects of curcumin on osteoclasts (cells which resorb bone) and osteoblasts (cells responsible for bone formation) have previously been investigated *in vitro*[8–12] and *in vivo*[13–16].

Nitric oxide (NO) is a gaseous molecule generated from l-arginine during the catalization of nitric oxide synthase (NOS), and it plays crucial roles in catalization and in the nervous, cardiovascular and immune systems [17]. Three isoforms of NOS are recognized: neural NOS (nNOS or NOS-1), inducible NOS (iNOS or NOS-2) and endothelial NOS (eNOS or NOS-3) [18]. Human osteoblasts have been shown to express both iNOS and eNOS [19]. Several agonists, such as proinflammatory cytokines and bacterial lipopolysaccharides, increase iNOS expression, whereas eNOS activities are induced by stimulators, such as estradiol and estrogen, indicating that iNOS and eNOS may play a role in bone inflammation and physiology [19,20]. The exact mechanism(s) by which NO regulates bone formation remain unclear. It has been proposed that NO directly induces osteoblast proliferation [21], while others have demonstrated that NO stimulates osteoblast proliferation via the induction of prostaglandin E2 (PGE2) production [22]. Evidence from gene knock-out studies has shown that bone formation is, at least in part, mediated by nitric oxide (NO), because mice deficient in endothelial nitric oxide synthase (eNOS) and mice deficient in the eNOS downstream effector (cGMP)-dependent protein kinase (PKG) show bone abnormalities. Additionally, inducible NOS (iNOS)-null mice also show imbalances in bone osteogenesis and abnormalities in bone healing [23]. Curcumin has been widely described to inhibit iNOS expression and NO production, at least in part via direct interference in NF-κB activation [24,25]. Therefore, the aim of the present study was to test the hypothesis that the exposure of human osteoblast-like cells to curcumin may affect growth and mineralization by a mechanism partially regulated by NO.

## 2. Results and Discussion

### 2.1. Cell Proliferation and NO Levels

**Figure 1:**
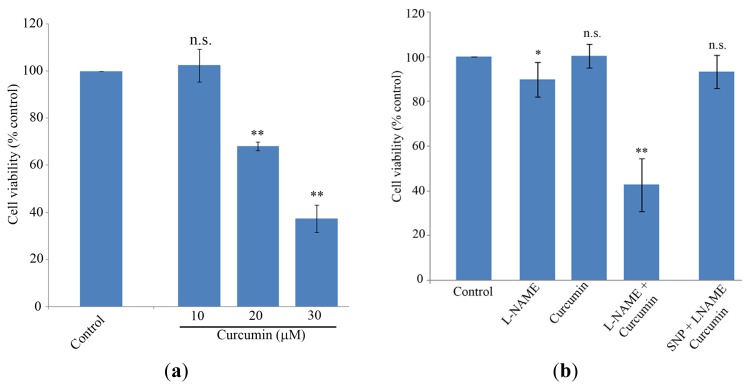
(**a**) Concentration-dependent effect of curcumin on MG-63 cells viability. Cells were incubated with various concentrations of curcumin for 24 h, after which cell viability was measured using MTT. Bars represent mean values, error bars represent SEM: n.s. (no significant difference compared to control cells), ** significant difference compared to control cells (*p* < 0.001). (**b**) Effect of curcumin (10 μM); L-NG-Nitroarginine Methyl Ester (L-NAME) (1 mM) and Sodium nitroprusside (SNP) (0.2 mM) at the indicated combinations after 24 h of exposure. Bars represent mean values, n.s. (no significant difference compared to control cells), * significant difference compared to control cells (*p* < 0.05), ** significant difference compared to control cells (*p* < 0.001).

Cell proliferation was measured using the MTT assay (Figure 1a,b). Curcumin significantly decreased cell viability in a dose-dependent manner in MG-63 cells. A dose of 20 μM of curcumin decreased cell viability to below 80% after 24 h of culture and 30 μM of curcumin decreased viability to below 50% (*p* < 0.001). No significant differences were found after 10 μM curcumin exposure (*p* > 0.05) (Figure 1a). Thus, 10 μM curcumin was selected as the working concentration for the rest of the experiments. At the tested concentrations, L-NAME moderately affected cell viability (*p* < 0.05) (Figure 1b), while the combination of L-NAME and curcumin substantially decreased cell viability after 24 h of culture (*p* < 0.001 *vs.* control cells) (Figure 1b). Supplementation of MG-63 cultures with 0.2 mM SNP prevented the cell death observed after co-stimulation with L-NAME and curcumin (*p* > 0.05 *vs.* control cells) (Figure 1b).

**Figure 2:**
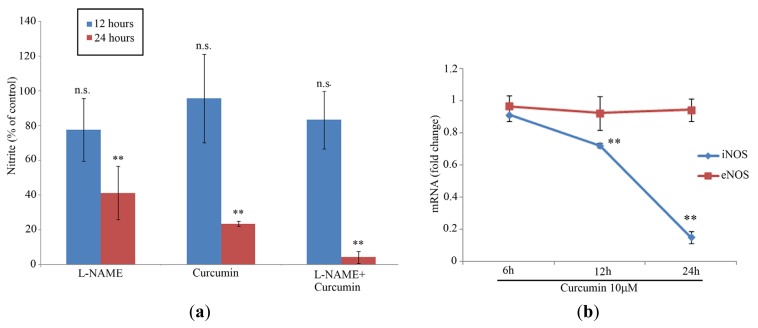
(**a**) Nitrite production by MG-63 cells at 12 and 24 h. Nitrite production was expressed as percentage of control (unstimulated cells). ** (*p* < 0.001), n.s. (not significant) *vs.* control cells in both cases. Bars represent mean values; error bars represent SEM. (**b**) Temporal changes in the expression of iNOS mRNA after curcumin (10 μM) exposure after 6, 12 and 24 h. Dots represent mean values; error bars represent SEM. ** Significant difference compared to control cells (*p* < 0.001).

As observed in Figure 2a, the nitrite concentration was measured in the supernatants of the cultured cells at 12 and 24 h post-stimulation. No significant difference *vs.* control cells was found at 12 h (*p* > 0.05 in all cases). After 24 h, cells cultured in the presence of either 1 mM L-NAME or 10 μM, curcumin had significantly decreased NO production (*p* < 0.001) (Figure 2a). Furthermore, the combination of both agonists abrogated NO production in MG-63 cells after 24 h of incubation (Figure 2a).

### 2.2. iNOS/eNOS Gene Expression

The results of the quantitative PCR analysis of MG-63 cells cultured in the presence of 10 μM curcumin are shown in Figure 2b. iNOS mRNA expression was significantly altered after 12 h of culture in the presence of 10 μM curcumin (*p* < 0.01). In contrast, the eNOS expression was not affected by incubation with curcumin (*p* > 0.05) (Figure 2b).

### 2.3. Mineralization

**Figure 3:**
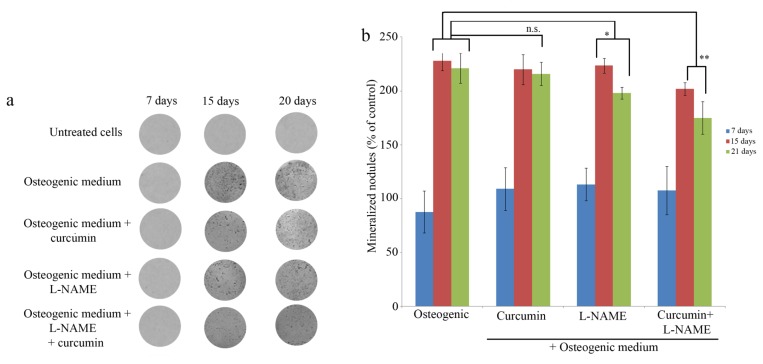
(**a**) Effect of curcumin (10 μM) and L-NAME (1 mM) on mineralized nodules in MG-63 cells. Cells were untreated or treated with osteogenic medium, osteogenic medium + curcumin, osteogenic medium + LNAME or osteogenic medium + curcumin and L-NAME for 21 days, and, then, mineralization was determined by AR-S staining. (**b**) Results are expressed as percentage of control. The data are expressed as mean ± SEM of three determinants. n.s. (no significant difference compared to control cells), * significant difference compared to control cells (*p* < 0.05), ** significant difference compared to control cells (*p* < 0.001).

From days 7–15, MG-63 cells exhibited significant AR-S staining in the presence of OM (Figure 3a). With increasing culture time, AR-S staining in the cells of the control group increased gradually. However, from days 15–21, AR-S staining was decreased in the experimental groups (Figure 3a). AR-S staining was used to quantify mineralized nodules. Curcumin did not decrease the rate of mineralization in MG-63 cells (*p* > 0.05 *vs.* OM). In contrast, L-NAME significantly decreased the number of nodules (*p* < 0.05 *vs.* OM), while the combination of both agonists greatly decreased the rate of MG-63 cell mineralization (*p* < 0.001 *vs.* OM) (Figure 3b).

### 2.4. COLI/OCN Gene Expression

**Figure 4:**
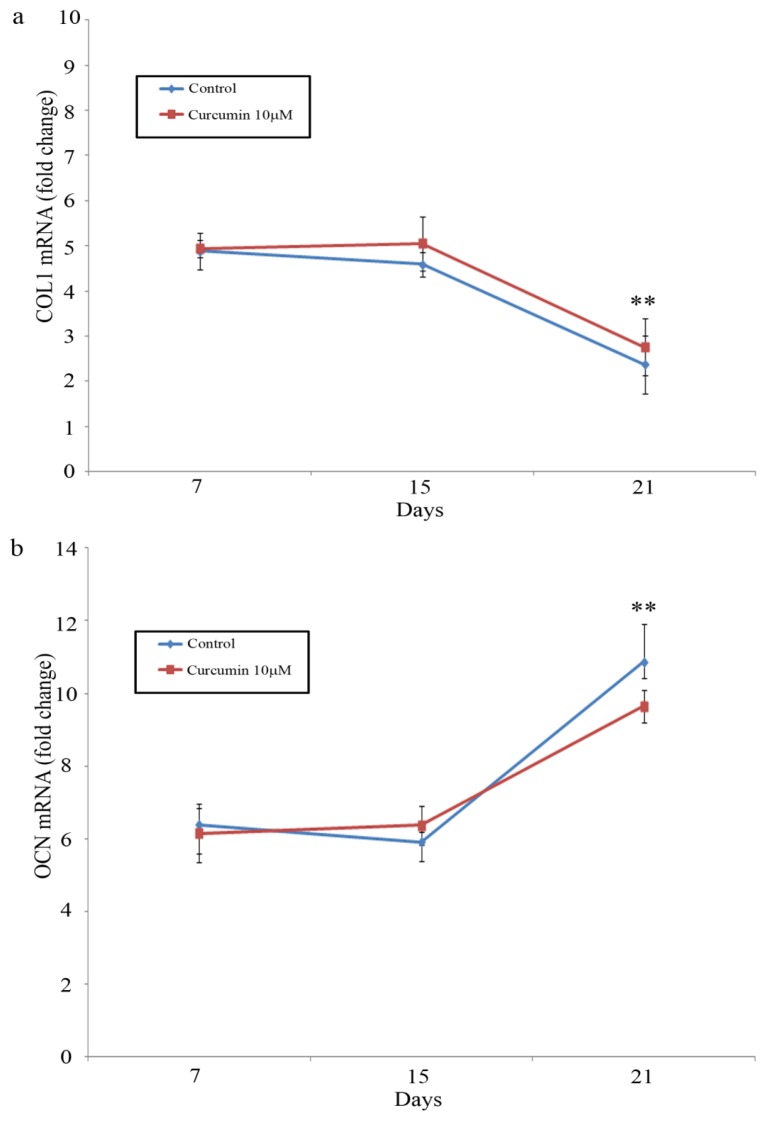
(**a**) Time course of alterations in COLI mRNA and OCN mRNA. (**b**) Concentration after the 7, 15 and 21 day of exposure to 10 μM curcumin + osteogenic medium or osteogenic medium alone. Total cellular RNA was extracted from MG-63 cells as described in Methods. COLI and OCN mRNA amount was determined by performing RT-PCR. Data represent the mean ± SEM for a minimum of three separate experiments. ** Significant difference compared to day 15 cells (*p* < 0.01). Not significant differences were found between curcumin stimulated cells and controls.

The results of the quantitative PCR assay of MG-63 cells cultured with either 10 μM curcumin or OM are shown in Figure 4. The rate of COLI mRNA expression remained constant and decreased from day 15 to day 21 of culture (*p* < 0.01). No significant differences were found in the rate of expression between control (OM) cells and curcumin-stimulated cells (*p* > 0.05) (Figure 4a). Similarly, the levels of OCN remained constant until day 15, when a significant (*p* < 0.01) increase was detected (Figure 4a). No significant differences were found between the rate of expression observed in control (OM) cells and curcumin-stimulated cells after 21 days of culture (Figure 4b).

### 2.5. ALP Activity and Gene Expression

**Figure 5:**
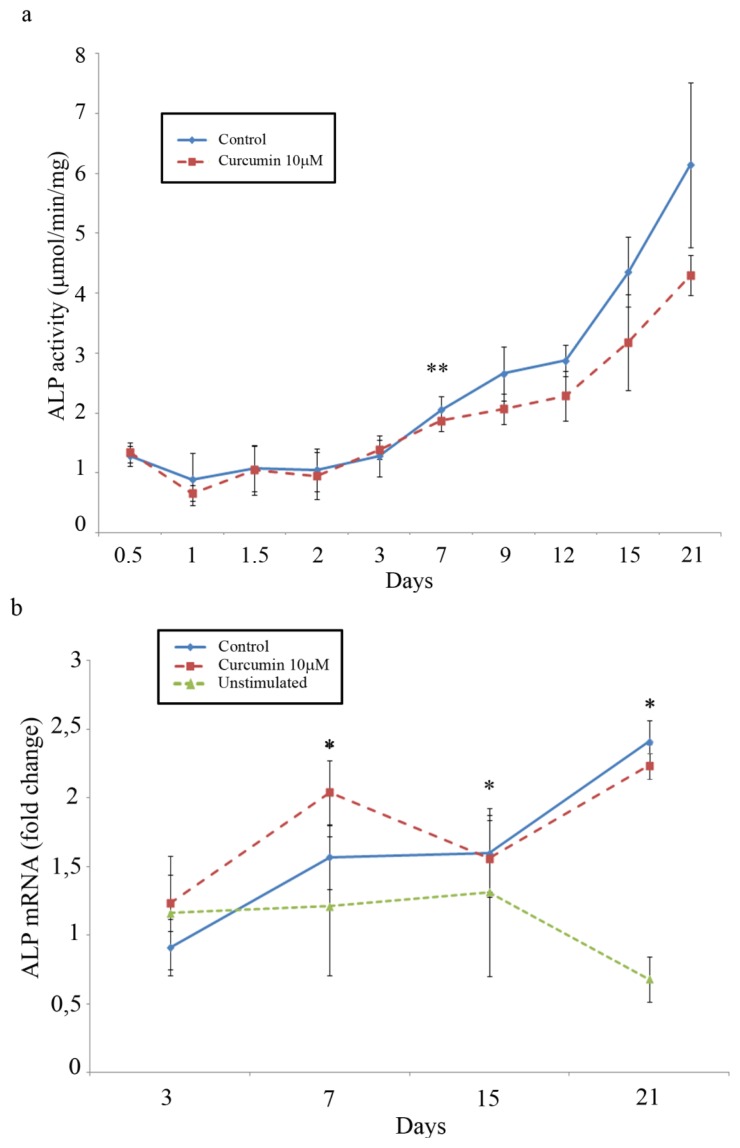
(**a**) The ALP activity of MG-63 cells at 0.5, 1, 1.5, 2, 3, 7, 9, 12, 15 and 21 post seeding in the presence of either osteogenic medium or osteogenic medium + curcumin 10 μM. ** (*p* < 0.001 *vs.* day 0.5 in both conditions). Significant differences (*p* < 0.001) were found from day 9 of culture to the end of the experiment between the experimental conditions. (**b**) Time course of alterations in ALP mRNA concentration after the 3, 7, 15 and 21 day of exposure to 10 μM curcumin + osteogenic medium, osteogenic medium or culture medium (unstimulated). Total cellular RNA was extracted from MG-63 cells as described in Methods. ALP mRNA amount was determined by performing RT-PCR. Data represent the mean ± SEM for a minimum of three separate experiments. * Significant difference compared to unstimulated cells (*p* < 0.05). Not significant differences were found between curcumin stimulated cells and osteogenic medium.

Figure 5a shows the ALP activity in MG-63 cells in the presence of OM or OM + 10 μM curcumin after the number of days indicated. ALP activity increased constantly from day 7 with culture time. Statistically significant differences were observed thereafter (*p* < 0.001 *vs.* 0.5 days). Significant differences between the experimental conditions were found from day 9 after culture to the end of the experiment (*p* < 0.001). In all cases, cells cultured in the presence of 10 μM curcumin exhibited lower ALP activity than the OM group (Figure 5a).

The results of the quantitative PCR assay of MG-63 cells cultured with either 10 μM curcumin or OM at the indicated times are shown in Figure 5b. Significant differences in ALP mRNA expression were observed between cells cultured in the presence of OM or OM + 10 μM curcumin *vs.* unstimulated cells at 7, 15 and 21 days of culture (*p* < 0.05). No significant differences were found between OM and OM + 10 μM curcumin at any time point tested (*p* > 0.05) (Figure 5b).

### 2.6. Discussion

Curcumin is a natural polyphenol extracted from *Curcuma longa*. Curcumin exhibits potent antioxidant and anti-inflammatory activity, inhibiting the production of free radicals, promoting radical scavenging and suppressing the production and release of inflammatory mediators. These properties may facilitate the attenuation of tissue and cellular injury in the liver, heart, kidney, brain and bone, induced by oxidation or inflammation. Curcumin has a long history of use as a medicinal compound and is known to also have multiple anti-cancer properties. It also affects a broad range of cellular targets at the molecular level, including STAT3 [26], Wnt/β-catenin [27], NF-κB [28] and HER2 [1], and the proteasome [29]. Curcumin also induce apoptosis regulated by multiple signaling pathways, induce or accentuate apoptosis indirectly by sensitizing cells to chemotherapeutical drugs and some contrary studies show that curcumin is also able to inhibit apoptosis [30].

In the present study, we showed that the treatment of MG-63 cells with curcumin inhibited their proliferation. This result is consistent with previous studies performed in human [9] or rat [10] osteoblast and with others, indicating that curcumin inhibited the proliferation of several cell types, most likely due to an apoptosis-dependent mechanism [31]. This mechanism has not been completely elucidated, but it may be, at least in part, due to the ability of curcumin to suppress the activation of AP-1, a dimeric transcription factor consisting of a Fos-related protein and a Jun-related protein as recently described [32].

In recent years, the beneficial effect of NO in bone tissue has received particular attention. However, the mechanism underlying the protective effect of NO against bone damage has not been fully elucidated. The impact of NO on osteoblast metabolism is bi-directional. Under physiological conditions, NO derived from eNOS is an important local mediator and second messenger for systemic hormones, including calcitonin gene-related peptide, parathyroid hormone and sex steroids, particularly estrogen, which take part in the regulation of bone function [33]. However, excess local production of NO (as observed with the use of NO donors) induces cytotoxicity in osteoblastic cells [34]. Thus, a biphasic role has been attributed to NO in osteoblast activity. *In vitro* studies have shown that the small amount of NO produced constitutively by osteoblasts might act as an autocrine stimulator of osteoblast growth and differentiation, while high NO concentrations inhibit cellular proliferation without affecting differentiation. Recent evidence suggests that high NO concentrations may exert at least a pro-apoptotic effect on osteoblasts and that this is mediated in part by cGMP [35]. While some investigators have shown that slow-release NO donors stimulate osteoblast growth and differentiation *in vitro*[36], other researchers report that NO donors and NOS inhibitors have little effect on osteoblast growth or differentiation, except at high concentrations, where inhibitory effects were observed [37].

Thus, the observed cellular response appears to depend, in some manner, on the NO concentration. In the present study, the cellular nitric oxide level was measured using the Griess reaction. The data demonstrate that NO secretion by curcumin-treated cells is significantly lower than unstimulated cells. To further examine the role of endogenous NO formation, we used a pharmacological approach to interfere with this pathway at two levels: (1) L-NAME, which specifically inhibits NOS activity and blocks signal transduction from NOS to NO; and (2) curcumin, which downregulates iNOS expression. As L-NAME has an effect on arginine metabolism, it could in fact affect protein synthesis and, consequently, the mineralization process in MG-63 cells. In this sense, it has been described that L-NAME weakly affects arginine availability and arginine transport activity in MG-63 cells [38].

Together, curcumin and L-NAME successfully abolished the NO levels in MG-63 cells. NO plays a fundamental role in the differentiation of osteoblasts, and it acts as an important second messenger in the signaling process that leads to extracellular matrix maturation, activating soluble guanylyl cyclase to produce cGMP, which in turn stimulates protein kinase G [39].

Osteoblast differentiation comprises three distinct processes: proliferation, maturation of the extracellular matrix and mineralization. During their developmental sequence, osteoblasts express genes associated with differentiation. ALP is important in the extracellular matrix maturation period, during which it forms calcified nodules. We observed that curcumin can modulate the differentiation of MG-63 cells, which was also indicated by differentiation markers ALP, OCN and COLI and the mineralization study (in combination with L-NAME). We observed changes in the early stages, with increased ALP expression in curcumin-stimulated cells at seven days post-culture. However, there was a significant decrease in the late stages of osteoblast differentiation at day 21, evidenced by the decelerating accumulation of calcium in osteoblasts. We failed to detect changes in the expression of OCN and COLI in the experimental groups, which might indicate that curcumin does not affect their mRNA expression, although it has been proposed that NO might enhance OCN gene expression through the cAMP response element activated by cGMP [40]. However, lower ALP activity was detected in curcumin-stimulated MG-63 cells, confirming an effect on osteoblast differentiation. ALP is a biomarker used to evaluate bone metabolism; staining methods are also commonly used to visualize bone nodule formation. The mineralization study revealed significant differences in the number of calcified bodies in curcumin-treated cells that significantly decreased when curcumin was combined with the NOS inhibitor L-NAME. This *in vitro* evidence suggests that NO may have a role in the mechanism leading to MG-63 cell differentiation to osteoblasts as proposed by others [19].

Not surprisingly, the osteogenic differentiation marker levels in the curcumin-treated groups were significantly lower than in the unstimulated cultures. These results suggest that NO itself or cGMP (latter produced by NO signaling) might promote osteoblastic metabolism and that its inhibition affects it. Additionally, it has recently been proposed that curcumin and curcumin analogs play a positive role in the differentiation of mouse osteoblasts by stimulating Smad signaling and suppressing NF-κB activation [41,42]. The inhibition of NF-κB signaling and the subsequent expression of iNOS and other molecules has been widely described in several cell lines and culture conditions [24].

As a limitation of our study, we did not address the signaling pathways mediated by NO in MG-63 cells or whether the inhibition of its expression is sufficient to trigger the inhibition of the pathway’s downstream elements, which may affect the process of osteogenesis. Further studies are needed to clarify these issues.

## 3. Experimental Section

### 3.1. Tissue Culture

A human osteoblast-like cell line, MG-63, was used as the osteoblasts in this study. The cells were maintained in a medium consisting of Dulbecco’s modified Eagle’s medium (DMEM) supplemented with 10% (*v*/*v*) heat-inactivated fetal calf serum (Invitrogen Gibco Cell Culture Products, Carlsbad, CA, USA), 2 mM glutamine, 100 units/mL penicillin G, 100 μg/mL streptomycin sulfate and 2.5 μg/mL amphotericin B (Sigma, St. Louis, MO, USA). Cultures were kept at 37 °C in a humidified atmosphere of 95% air and 5% CO_2_. Upon reaching 70% confluence, cells were detached from the culture flask with a solution of 0.05% trypsin (Sigma, St. Louis, MO, USA) and 0.02% ethylenediaminetetraacetic acid (EDTA) (Sigma, St. Louis, MO, USA).

### 3.2. Cell Proliferation

Cell proliferation was determined by the MTT method. Osteoblasts were seeded at 1 × 10^4^ cells/mL per well in a 96-well plate without FBS and cultured at 37 °C for 24 h. Subsequently, the medium was replaced with DMEM containing either curcumin (dissolved in ethanol) at the indicated doses (10, 20 or 30 μM), 1 mM L-N^G^-nitroarginine methyl ester (L-NAME) or 0.2 mM sodium nitroprusside (SNP) (both dissolved in DMEM) (all from Sigma, St. Louis, MO, USA). At the end of the treatments (24 h), the medium was replaced with DMEM containing 0.5 mg/mL MTT (Sigma) and incubated for 4 h. Cellular reduction of the MTT tetrazolium ring resulted in the formation of a dark-purple water-insoluble deposit of formazan crystals. After incubation, the medium was aspirated, and DMSO was added to dissolve the formazan crystals. Absorbance was measured at 570 nm with a spectrophotometer.

### 3.3. Determination of Nitrite/Nitrate (NOx) Concentrations

Nitrite production was measured using the Griess reaction. After a 10 min incubation at 37 °C in the dark, absorbance was measured at 540 nm. A blank was prepared in the absence of cells, and its absorbance was subtracted from the sample absorbance. Sodium nitrite was used as a standard to build a calibration curve. Nitrite concentration was expressed as a percentage of the unstimulated cell nitrite concentration.

### 3.4. RNA Purification

RNA was purified from cells (5 × 10^6^) used in various experiments. Total RNA was isolated using the TRI REAGENT kit according to the manufacturer’s instructions (Sigma, St. Louis, MO, USA). Briefly, 1 mL of TRI REAGENT was added directly to the culture dish. The cell lysates were transferred by pipetting to an Eppendorf tube and incubated for 5 min at room temperature, and 0.2 mL of chloroform per 1 mL of TRI REAGENT was added to the tubes. The tubes were vortexed for an additional 15 min and incubated for 15 min at room temperature (RT). The resulting mixture was centrifuged at 12,000*g* for 15 min. The aqueous phase was transferred to a fresh tube, and 0.5 mL of isopropanol was added. Samples were incubated for 10 min at RT and centrifuged at 12,000*g* for 15 min. Finally, the supernatant was removed, and the RNA pellet was washed twice with 1 mL 75% ethanol and once with 1 mL of 100% ethanol.

### 3.5. Quantitative Reverse Transcription-Polymerase Chain Reaction (RT-PCR) for iNOS, eNOS, Type I Collagen (COLI), Alkaline Phosphatase, Osteocalcin (OCN) and β-actin Genes

Total RNA (5 μg) was used as a template to synthesize cDNA using a Moloney Murine Leukemia Virus First Strand cDNA Synthesis Kit (Gene Choice, Frederick, MD, USA). The reaction buffer contained random hexamer primers and deoxynucleotide triphosphates (dNTPs). Aliquots of the cDNA product were used as the template for quantitative real-time polymerase chain reaction (RT-PCR) analysis, which was performed using an Applied Biosystems 7300 Real-Time PCR System (Foster City, CA, USA) and SYBR Premix Ex Taq (Takara Bio Inc., Shiga, Japan). The relative gene expression levels were normalized to the amount of β-actin mRNA. The primer sequences were as follows: human b-actin, 5′-CCACACCTTCTACAATGAGC-3′ (upstream) and 5′-CTCGTAG ATGGGCACAGTGT-3′ (downstream); human type I collagen alpha I, 5′-AAGATGTGCCACTCTG ACTG-3′ (upstream) and 5′-ATAGGTGATGTTCTGGGAGG-3′ (downstream); human alkaline phosphatase, 5′-CATCTGGAACCGCACGGAAC-3′ (upstream) and 5′-GCCTGGTAGTTGTTGTGA GC-3′ (downstream); human osteocalcin, 5′-CTCACACTCCTCGCCCTATT-3′ (upstream) and 5′-CAACTCGCACAGTCCGGAT-3′ (downstream); human eNOS, 5′-CCTGGAGAATGAGCAGA AGG-3′ (upstream) and 5′-GATGCTGTTGAAGCGGATCT-3′ (downstream); and human iNOS, 5′-TCTTCGAAATCCCACCTGAC-3′ (upstream) and 5′-TTTTCCAGGCCTCTACCTGA-3′ (downstream).

### 3.6. Alkaline Phosphatase (ALP) Activity

For the ALP activity assay, the cells were incubated with 4 mg/mL of a ρ-nitrophenyl phosphate solution (Sigma, St. Louis, MO, USA) at 37 °C for 30 min. ρ-Nitrophenol (PNP) production in the presence of ALP was measured by monitoring the solution absorbance at 405 nm using a spectrophotometer. The protein content of the extracts was measured using the Bio-Rad DC protein assay, and ALP activity was expressed as μmol/min/mg protein.

### 3.7. Mineralization

Alizarin red-S (AR-S) staining is a common histochemical technique used to detect calcium deposits in mineralized tissues and cultures, and positive AR-S staining for calcium has been shown to represent calcium phosphate and osteoblast culture mineralization. Control group cells were incubated in osteogenic medium (OM) containing 10% FBS, 50 g/mL ascorbic acid and 10 mM 2-glycerophosphate. The experimental groups were incubated with 10 μM curcumin, 1 mM L-NAME or both. After incubation for 21 days, cells were collected and stained with alizarin red. The cells were washed with PBS and fixed in 70% ethanol for 1 h at 4 °C. After additional washing in PBS, they were incubated in 0.4% alizarin red-S in water for 10 min at room temperature, followed by a final incubation in PBS for 15 min. The stained cells were photographed and then subjected to a quantitative destaining procedure using 10% (*w*/*v*) cetylpyridinium chloride in 10 mmol L^−1^ sodium phosphate (pH 7.0) for 30 min at room temperature. Absorbance was measured at 562 nm on a spectrophotometer, and the results were expressed as a percentage of the control (unstimulated) cell absorbance.

### 3.8. Data Analysis

The results were expressed as the means ± SEM. Statistical analyses were performed with ANOVA and Fisher’s exact test, when appropriate. The P value cutoff for statistical significance was defined as *p* < 0.05. For the single agonist experiment, the differences between group means were evaluated by Student’s *t* test (SPSS statistical package, version 19.0, IBM-SPSS: Chicago, IL, USA, 2010). Differences were considered statistically significant when *p* < 0.05.

## 4. Conclusions

In conclusion, curcumin exposure of MG-63 cells modulates osteoblastic differentiation by a mechanism partially related to the inhibition of NO production.

The results described in this report can be summarized as follows. (1) The treatment of MG-63 cells with curcumin abrogated iNOS expression and decreased NO levels. (2) Treatment with both curcumin and L-NAME decreased NO levels, inhibiting cell proliferation. This effect was prevented by the NO donor SNP, thereby indicating a role for NO as a regulatory factor in MG-63 proliferation. (3) Under osteogenic conditions, the incubation of MG-63 cells with both curcumin and L-NAME decreased the level of mineralization, indicating that NO plays a role in the osteoblastic profile of MG-63 cells.
